# Uric acid, ferritin and γ-glutamyltransferase can be informative in prediction of the oxidative stress

**DOI:** 10.3164/jcbn.18-23

**Published:** 2019-01-16

**Authors:** Kanae Oda, Emiko Kikuchi, Emiko Kuroda, Chizumi Yamada, Chiori Okuno, Nana Urata, Noriaki Kishimoto, Akira Kubo, Naoaki Ishii, Yasuhiro Nishizaki

**Affiliations:** 1 Department of Clinical Health Science, Tokai University School of Medicine, 1-2-5 Yoyogi, Shibuya-ku, Tokyo 151-0053, Japan; 2 Tokai University Tokyo Hospital, 1-2-5 Yoyogi, Shibuya-ku, Tokyo 151-0053, Japan; 3 Tokai University Hospital, 143 Shimokasuya, Isehara-shi, Kanagawa 259-1193, Japan

**Keywords:** anti-aging health check-ups, oxidative stress-related marker, uric acid, ferritin, γ-glutamyltransferase

## Abstract

The anti-oxidant system is affected not only by aging but also many lifestyle factors. We aimed to clarify the determinants of medical check-up items affecting the anti-oxidant system. We studied 959 Japanese individuals who underwent anti-aging health check-ups (mean age: 61.1 years) at Tokai University from 2006 to 2016. As parameters of oxidative stress, we measured serum total anti-oxidant status, 8-hydroxy-2'-deoxyguanosine, and isoprostane. Anti-aging health check-up data and lifestyle information were collected from participants in this study. Step-wise multiple regression analyses were conducted to identify determinants that influence serum total anti-oxidant status, 8-hydroxy-2'-deoxyguanosine, and isoprostane, respectively. Serum total anti-oxidant status was significantly correlated with uric acid, vitamin A, folate, and valine. 8-hydroxy-2'-deoxyguanosine was significantly correlated with age, ferritin, drinking habit, and vitamin Eα. Isoprostane was significantly correlated with vitamin Eα, γ-glutamyltransferase, ferritin, and smoking habit. The strong antioxidant powers of uric acid and vitamins were confirmed. It was suggested that branched-chain amino acids themselves such as valine or peptides containing them may possess antioxidant ability because of its strong correlation. Uric acid, ferritin, and γ-glutamyltransferase, which are common items measured in medical checkups, can be informative in predicting the oxidative stress situation in a general medical examination.

## Introduction

Humans consume oxygen to obtain the energy needed for sustaining life, but the process of metabolizing oxygen produces reactive oxygen species (ROS), which causes bodily harm. Antioxidant systems to remove ROS from our bodies do exist, but these systems fluctuate not only with age but also under the impact of many lifestyle habits. If too much ROS accumulates in the body and causes oxidative stress, DNA, lipids, proteins, enzymes and other biopolymers in the body that are important for sustenance of life suffer oxidative damage. This can lead to cancer and a variety of other disorders such as lifestyle diseases and neurodegenerative diseases, and promotes aging.^(1)^

According to the 2017 Annual Report on the Aging Society released by the Cabinet Office (Japan), more than 27% of Japan’s population today are aged 65 or over, and by 2065 the percentage will be closer to 40%.^(2)^ In order to stay healthy for longer as we approach this “aged society,” how we control oxidative stress is an important issue. Accurately evaluating oxidative stress in the body and taking steps to reduce it are expected to be useful in preventing disease and controlling aging.

For more than ten years now, the Health Screening Center of Tokyo Hospital, Tokai University School of Medicine has been focusing attention on the oxidative risk caused by aging and lifestyle habits. We have been accumulating data on this issue in our “anti-aging health check-ups,” in operation since 2006.^(3)^ We have also been researching how health is impacted and whether a healthy life span can be extended by improving lifestyle habits without depending on supplements, and thereby avoiding oxidation risks.

Serum total antioxidant status (STAS), a water-soluble oxidant substance in blood, has been measured as an indicator for understanding antioxidant capacity against oxidative stress.^(4)^ 8-hydroxy-2'-deoxyguanosine (8-OHdG) is known as an indicator of DNA damage caused by oxidative stress,^(5)^ while 8-iso-Prostaglandin F2α (isoprostane), a product arising when phospholipids that serve an important function in the body are subjected to oxidative stress, is known as a marker of lipid system oxidative damage.^(6)^ In this research, particular attention was focussed on three of the oxidative stress-related markers measured in anti-aging health check-ups—STAS, 8-OHdG and isoprostane—in order to study factors that impact the oxidant-antioxidant balance. We studied the correlation between these and various items measured in anti-aging health check-ups, as well as information given by medical questionnaire.

## Materials and Methods

### Study participants

The participants were 959 patients (526 males, 433 females, ages 27–89, mean age 61.1 years) who had undergone anti-aging health check-ups between June 2006 and March 2016 at the Health Screening Center of the Tokai University School of Medicine. Those who were being treated for hypertension, diabetes or dyslipidemia were excluded from the study (Table 1).

### Statistical analysis

First, each of STAS, 8-OHdG and isoprostane, and items measured in general health check-ups at the hospital, were subjected to correlation analysis. Here, factors recognized as having a significant correlation (*p*<0.005), vitamins previously reported as antioxidants (vitamin A, vitamin C, vitamin Eα, β-carotene, vitamin B12 and folic acid),^(7)^ amino acids (valine, methionine, isoleucine, leucine, tyrosine, phenylalanine, lysine, histidine and arginine), age, gender, smoking habit, drinking habit and exercise habits were used as candidates for explanatory variables. Multiple regression analysis (step-wise method) was then carried out using STAS, 8-OHdG and isoprostane as the target variables, respectively. The category variables gender, smoking habit, drinking habit and exercise regime were analyzed using dummy variables for each, namely Male: 1, Female: 0, Smokes: 1, Does not smoke: 0, Does not drink: 0, Drinks a little: 1, Drinks at least 360 ml each time and on at least five days a week: 2, and Exercises: 1, Does not exercise: 0. As a result of studying multiple collinearity between the explanatory variables, a strong multiple collinearity was observed between the branched-chain amino acids (BCAA) valine, leucine and isoleucine and between erythrocyte count, hemoglobin and hematocrit. Therefore, correlation analysis was conducted on each target variable, and the variable with the highest correlation coefficient was adopted as a representative candidate for explanatory variables. Of BCAA, valine was used for STAS and leucine for isoprostane and 8-OHdG. Of erythrocyte count, hemoglobin and hematocrit, hemoglobin was used for STAS and erythrocyte count for isoprostane. No significant correlation with 8-OHdG was recognized in any of these. SPSS Statistics Ver.22 (IBM Corp, Armonk, NY) was used for all analyses, and the significant level was set at 0.05.

### Ethical consideration

The participants gave their consent for this research by signing a “Consent form for handling of personal information, etc.” when receiving their health check-ups. They were notified in writing that their anonymity was protected and that no information that could be used to identify specific individuals taking part in the research would be made public in publication of the research results. In our analysis, we used a database with the personal information removed. We complied with the “Ethical Guidelines for Epidemiological Research.” This research was conducted with the approval of the Ethics Review Board of the Tokai University School of Medicine.

## Results

### Correlation analysis

Table 2 shows the results of correlation analysis. STAS showed a significant correlation with height, abdominal girth, hemoglobin, total protein, albumin, HbA1c, uric acid and creatinin, with Pearson correlation coefficients of 0.160, 0.179, 0.250, 0.267, 0.331, 0.399, 0.441 and 0.286, respectively. 8-OHdG showed a significant correlation with ferritin and alkaline phosphatase (ALP), with Pearson correlation coefficients of 0.131 and 0.131, respectively. Isoprostane showed a significant correlation with LDL cholesterol, erythrocyte count, ferritin, total protein, γ-GT and ALP, with Pearson correlation coefficients of −0.183, −0.128, 0.137, −0.158, 0.138 and −0.163, respectively. Note that, because creatinin-corrected values were used for 8-OHdG and isoprostane as target variables in this research, creatinin was removed from the candidate explanatory variables for 8-OHdG and isoprostane.

### Multiple regression analysis

Table 3 shows the results of multiple regression analysis. Four explanatory variables (uric acid, folic acid, vitamin A and valine) were selected in the multiple regression analysis with STAS as a target variable, and all were observed to have a positive correlation (standard β was 0.541, 0.138, 0.138 and 0.112, respectively). Adjusted R-squared showing model conformity was 0.463 (*p*<0.0001).

There were four explanatory variables for 8-OHdG (age, ferritin, drinking habit and vitamin Eα), these showing a positive correlation with age and ferritin (standard β 0.202 and 0.173, respectively) and a negative correlation with drinking habit and vitamin Eα (standard β −0.173 and −0.136, respectively). Adjusted R-squared was 0.100 (*p*<0.0001).

There were four explanatory variables for isoprostane (vitamin Eα, γ-GT, ferritin and smoking habit), showing a negative correlation with vitamin Eα (standard β −0.217) and a positive correlation with γ-GT, ferritin and smoking habit (standard β 0.128, 0.119 and 0.100. respectively). Adjusted R-squared was 0.079 (*p*<0.0001).

## Discussion

Our bodies are constantly exposed to a wide variety of oxidative stresses. The degree of these stresses is greatly impacted not only by aging but also by various lifestyle habits. It is therefore important to know the levels of these individual oxidative stresses and avoid the risks associated with them in order to live a long and healthy life.

STAS is useful as a marker for inferring total oxidative stress; the higher it is, the higher the antioxidant capacity appears to be.^(4)^ In this research, four substances (uric acid, folic acid, vitamin A and valine) were chosen as variables that have a significant correlation with STAS. All of them showed a positive correlation, suggesting that they could have the effect of boosting antioxidant capacity. Uric acid showed a particularly high correlation (standard β 0.541), with an outstandingly high *t* value of 11.766 (*p*<0.0001) expressing the level of effect. Uric acid has previously been reported to have a strong antioxidant capacity,^(8)^ and this strong antioxidant capacity seems to have been reconfirmed this time. We think that the reason uric acid showed a correlation only with STAS is that both uric acid and STAS are water-soluble. On the other hand, it is reported that when uric acid is taken in the cells, it activates an NADPH oxidase and increases intracellular ROS.^(9)^ The intracellular uric acid may act on pro-oxidation. There is a possibility that uric acid showed no correlations with 8-OHdG, oxidative damage index for genes, and with isoprostane, for lipids of cell membranes, because of its bilateral character, antioxidation and pro-oxidation. It is also well-known that the negative effects that high levels of uric acid bring extend over a whole range of lifestyle diseases including ischemic heart disease as well as gouty arthritis, gout kidney and urinary stones.^(10–13)^ Thus, how to maintain uric acid values at appropriate levels—not too high, not too low—is expected to be a task for preventive medicine in future.

8-OHdG is an oxidized derivative of deoxyguanosine (dG), and a marker of DNA oxidative damage. As this dG has low oxidation reduction potential and is easily susceptible to oxidation by ROS, 8-OHdG is widely used today as a marker of oxidative stress that sensitively reflects the impact of ROS on the body.^(14,15)^

Because 8-OHdG shows the degree of damage to DNA, the higher the value is, the worse oxidation damage is. In this research, it showed a positive correlation with age and ferritin and a negative correlation with drinking habit and vitamin Eα. A correlation between age and 8-OHdG has also been reported in previous research,^(16,17)^ and this time results consistent with those studies were obtained. Drinking habit shows a negative correlation with 8-OHdG. Because drinking habit was analyzed by scoring “Does not drink” as 0, “Drinks a little” as 1, and “Drinks heavily” (drinks at least 360 ml each time on at least five days a week) as 2, this means that the more the subject drinks, the more the correlation moves toward antioxidation. A collective attribute of anti-aging health check-up patients who took part in this research is that they are all very health-conscious; more than half of them (54%) were classified as “non-drinkers.” Even among the “drinkers,” there are very few heavy drinkers (just a few %); in most cases they tended to enjoy red wine, Japanese sake or similar in moderation. Although no analysis was undertaken based on different types of alcoholic beverage this time, it is likely that they willingly drink red wine or Japanese sake, which have been reported to have an antioxidant capacity.^(18,19)^ This is thought to have affected the results this time.

Ferritin is an iron-storage protein that exists in all cells; it exchanges iron with transferrin and serves to maintain the amount of iron in the blood. Serum ferritin normally reflects stored iron. Low ferritin values are useful in knowing states of iron deficiency such as iron deficiency anemia, while high ferritin values indicate states of iron overload such as hemochromatosis.^(20)^ On the other hand, hyperferritinemia does not always reflect iron overload. It is reportedly related to hepatitis and other inflammatory diseases, malignant tumors and myocardial infarction.^(21–23)^ A feature common to all of these is cell disintegration, and it is thought that ferritin existing inside cells flows into the blood, causing serum ferritin levels to rise. During inflammation, moreover, ferritin synthesis is also promoted by TNFα and other inflammatory cytokines,^(24–26)^ and this is also thought to cause a rise in ferritin. Ferritin, used as an inflammatory marker, is also reported to be actively involved in the production of ROS in the locus of inflammation.^(16)^ Ferritin causes increased production of superoxides by neutrophils, iron ions transformed from trivalent to divalent act as catalysts of a Harber-Weiss reaction, and hydroxy radicals are produced. Hydroxyl radicals are strong ROS that cause DNA damage and various other types of oxidative stress.^(27,28)^ In this research, strong correlations between ferritin and two oxidative stress markers (8-OHdG and isoprostane) have been recognized. This is thought to corroborate the reaction leading to increased ROS production by ferritin itself.

Isoprostane is a substance formed through oxidation of phospholipids existing in cell membranes and lipoproteins by free radicals, and links to smoking, diabetes and arteriosclerosis have been reported.^(29–31)^ The higher the isoprostane value, the higher the degree of oxidation is thought to be. In this research, a positive correlation with smoking habit and γ-GT was recognized. It has been reported in many studies that smoking is a cause of ROS generation, and the positive correlation with isoprostane could be seen to corroborate previous reports. We wonder why the correlation with other markers would not be seen. Probably the low smoking rate (14%) of this study may influence on the result. Isoprostane is known as the sharp oxidation damage index that is easier to be oxidized than a nucleic acid or a protein. In the subjects of this study that had such low smoking rate, there is a posibility that only the sharper index “isoprostane” might show a correlation with smoking. γ-GT is known as a sensitive marker of liver disorders. Its function is an enzyme that hydrolyzes a γ-glutamyl compound or transfers the γ-glutamyl group to a peptide or an amino acid. Glutathione, known as a strong antioxidant, is a type of γ-glutamyl peptide; when hydrolyzed by γ-GT, it is broken down into glutamic acid and cysteinyl glycine. Paolicchi reported that γ-GT is involved in the production of oxidant LDL cholesterol.^(32)^ Elevation of serum γ-GT activity is thought to be related to an increase in oxidative stress. The fact that isoprostane and γ-GT showed positive correlations in this research suggests that γ-GT is useful as an oxidative stress marker.

Vitamins have previously been reported to have a strong antioxidant capacity.^(33,34)^ We found that folic acid and vitamin A showed a significant correlation with STAS, and vitamin Eα with 8-OHdG and isoprostane, each moving toward antioxidation. It was suggested that ingesting these vitamins daily in food or via supplements could be effective in raising their own antioxidant capacity and reducing oxidative stress inside the body.

Also this time, the branched-chain amino acid valine was shown to have a significant positive correlation with STAS. Since strong multiple collinearity was recognized between three branched-chain amino acids, we used valine to represent branched-chain amino acids as a candidate explanatory variable, based on the result of a single correlation this time. However, leucine and isoleucine also show significant positive correlation with STAS in correlation analysis, respectively, and all branched-chain amino acids are thought to show positive correlation with STAS. It has been suggested that branched-chain amino acids act as antioxidants.^(35,36)^ In our research, the standard β of valine was 0.112, which is lower than that of uric acid and vitamins, but since the *p* value was sufficiently small and significant, the possibility of branched-chain amino acids acting as antioxidants was suggested. The amino acids used in the analysis this time were limited to nine in number—valine, methionine, leucine, isoleucine, tyrosine, phenylalanine, lysine, histidine and arginine—which could be measured reliably when the anti-aging health check-ups were started in 2006. Besides these, however, other amino acids have been reported to have strong antioxidant capacity. We have at present measured 40 different amino acids, and we would like to increase the number of amino acids to be analyzed by our studies in future.

The three models created this time all had sufficiently small *p* values, but the coefficients of determination for, 8-OHdG and isoprostane, in particular, were small. Many variables that impact the target variables are still thought to exist. In future, we would like to increase our data and conduct further study.

In this research, uric acid and vitamins were confirmed to have strong antioxidant capacity. Moreover, a positive correlation was recognized between STAS and valine as a representative of branched-chain amino acids, and the possibility was suggested that branched-chain amino acids themselves or the peptides they contain could have antioxidant capacity. Uric acid, γ-GT and ferritin have even been measured in general health check-ups, and this finding could be informative when predicting the state of oxidative stress in patients undergoing general check-ups.

## Author Contributions

KO designed the study, analyzed data, and wrote the manuscript with contributions and suggestions from all authors; EK, EK, CY, CO, NU, NK, AK, NI and YN collected data. All authors contributed to the discussion and interpretation of the results.

## Figures and Tables

**Table 1:** Study participants

	Male	Female
Health check-up period	June 2006–March 2016	
Participants	526	433
Age at time of check-up	Mean age 59.8 ± 11.1 years (27–84)	Mean age 62.2 ± 11.8 years (27–89)

**Table 2:** Results of correlation analysis between oxidative stress-related markers and general health check-up items

	STAS			8-OHdG			Isoprostane	
	Correlation coefficient	*p*		Correlation coefficient	*p*		Correlation coefficient	*p*
Height	0.160*	0.005		–0.094	0.053		0.056	0.327
Weight	0.102	0.073		–0.059	0.226		0.144	0.011
BMI	0.010	0.866		0.002	0.974		0.151	0.007
Girth	0.179*	<0.0001		0.003	0.942		0.065	0.125
Body fat percentage	–0.111	0.052		0.072	0.136		0.040	0.480
Systolic blood pressure	0.055	0.198		0.022	0.536		0.000	0.996
Diastolic blood pressure	0.103	0.015		–0.028	0.441		–0.002	0.963
LDL cholesterol	–0.035	0.406		–0.059	0.096		–0.183*	<0.0001
HDL cholesterol	–0.100	0.017		0.037	0.301		–0.019	0.654
Neutral fat	0.055	0.193		–0.068	0.057		–0.056	0.184
High sensitivity CRP	–0.009	0.829		–0.005	0.888		0.062	0.141
Leukocyte count	0.056	0.181		0.032	0.371		–0.015	0.729
Erythrocyte count	—	—		–0.018	0.613		–0.128*	0.002
Hemoglobin	0.250*	<0.0001		0.000	0.998		—	—
Hematocrit	—	—		–0.007	0.851		—	—
Platelets	–0.052	0.215		–0.088	0.013		–0.075	0.073
Serum iron	0.070	0.095		–0.025	0.488		0.070	0.094
Ferritin	0.116	0.006		0.131*	<0.0001		0.137*	0.001
Total protein	0.267*	<0.0001		0.050	0.231		–0.158*	0.003
Albumin	0.331*	0.002		0.039	0.494		0.257	0.015
AST	0.109	0.009		0.054	0.128		0.070	0.094
ALT	0.116	0.006		0.035	0.320		0.048	0.255
γ-GT	0.022	0.608		–0.054	0.132		0.138*	0.001
LDH	0.012	0.784		0.083	0.020		0.067	0.108
ALP	0.001	0.991		0.131*	0.002		–0.163*	0.002
CHE	0.157	0.154		–0.143	0.012		–0.128	0.237
Serum amylase	0.084	0.118		0.103	0.014		–0.087	0.105
Fasting blood glucose	0.093	0.026		–0.066	0.063		0.080	0.056
HbA1c	0.399*	<0.0001		–0.101	0.006		–0.053	0.221
Uric acid	0.441*	<0.0001		–0.039	0.276		–0.033	0.425
Urea nitrogen	0.012	0.827		0.005	0.909		0.053	0.326
Creatinin	0.286*	<0.0001		—	—		—	—

*p<0.005

**Table 3:** Results of multiple regression analysis on oxidative stress-related markers

Target variable	Explanatory variable	Parameter estimates						Model conformity	
		Estimate	SE	*t* value	*p*	Standard β		Adjusted R^2^	*p*
STAS	Uric acid	42.849	3.642	11.766	<0.0001	0.541		0.463	<0.0001
	Folic acid	1.646	0.470	3.504	0.001	0.138			
	Vitamin A	0.890	0.276	3.218	0.001	0.138			
	Valine	0.299	0.120	2.040	0.013	0.112			

8-OHdG	Age	0.052	0.009	5.644	<0.0001	0.202		0.100	<0.0001
	Ferritin	0.005	0.001	4.789	<0.0001	0.173			
	Drinking	–0.762	0.160	–4.770	<0.0001	–0.173			
	Vitamin Eα	–0.090	0.023	–3.857	<0.0001	–0.136			

Isoprostane	Vitamin Eα	–0.062	0.013	–4.722	<0.0001	–0.217		0.079	<0.0001
	γ-GT	0.004	0.001	2.703	0.007	0.128			
	Ferritin	0.002	0.001	2.573	0.010	0.119			
	Smoking	0.266	0.119	2.239	0.026	0.100			
